# Polynomial modelling of high-quality yet incomplete rare earth element data sets and a holistic assessment of REE anomalies

**DOI:** 10.1038/s41598-025-89227-2

**Published:** 2025-02-13

**Authors:** David M. Ernst, Joachim Vogt, Michael Bau, Malte Mues

**Affiliations:** 1 Critical Metals for Enabling Technologies – CritMET, School of Science, Constructor University, Campus Ring 1, 28759 Bremen, Germany; 2 School of Science, Constructor University, Campus Ring 1, 28759 Bremen, Germany; 3 Chair for Software Engineering, TU Dortmund University, Otto-Hahn-Straße 12, 44227 Dortmund, Germany

**Keywords:** Rare earth elements, Regression analysis, Monte Carlo simulation, Long tail data, Anomaly quantification, Computer science, Software, Geochemistry, Volcanology

## Abstract

Rare earth elements (REEs) are powerful proxies used in many (bio-)geochemical studies. Interpretation of REE data relies on normalised REE patterns and anomaly quantification, and requires complete data. Therefore, older, high-quality REE data determined by neutron activation or isotope dilution methods are often ignored, as they did not provide complete data. Similarly, modern analytical data can lack certain REEs due to quantification limits, interferences or usage of REE spikes. However, such data may be the only information available since sample material was consumed, sample locations became inaccessible, or samples represent past states of a dynamic natural system. Therefore, the ability to impute such high-quality data is of value for many geoscientific sub-disciplines. We use a polynomial modelling approach to impute missing REE data, verify the method’s applicability with a large data set (>13,000 samples; PetDB), and complement three originally incomplete REE data sets. Good fitting results (SD <6%) are supported by Monte Carlo simulations for assessing the model uncertainties (± 12%). Additionally, we provide a procedure to quantify REE anomalies, including uncertainties, which were usually not determined in the past but are essential for scientific comparison of REE anomaly data between different data sets. All Python scripts are provided.

## Introduction

The rare earth elements (REE) are a group of elements with similar physical properties and coherent geochemical behaviour. Therefore, REEs are widely used as proxies for numerous geochemical processes (e.g.^1–4^), physico-chemical environments or element sources^5–8^. Especially individual REE anomalies are of great scientific interest since they indicate deviations from “normal” conditions. For example, Eu anomalies can be indicative of reducing and hot (>250 °C) formation conditions (e.g.^9–11^). Research on REE intensified in the 1960s with the emergence of neutron activation analysis (NAA; e.g.^12–14^), and, subsequently, isotope dilution (ID; e.g.^15,16^). However, often, only incomplete REE data sets could be determined due to the specific limitations of the analytical method used. Isotope dilution techniques, for example, do not allow the determination of the monoisotopic REEs praseodymium (Pr), terbium (Tb), holmium (Ho), and thulium (Tm). Neutron activation datasets usually lack praseodymium, dysprosium (Dy), holmium, and erbium (Er). As the interpretation of REE data is predominantly based on shale- or chondrite-normalised REE (REE_N_) distribution patterns, missing REEs can alter the appearance of REE patterns and hence affect the interpretation or, as in the case of the lanthanide tetrad effect, even prevent it. Introduced in the 1970s, inductively coupled plasma atomic emission spectroscopy (ICP-AES) and later inductively coupled plasma mass spectrometry (ICP-MS) allowed the measurement of all 14 naturally occurring REEs (e.g.^17^). Although incomplete, the comparison of reference material (RM) data derived from ID and NAA measurements with ICP-MS measurements shows that ID and NAA are accurate and precise analytical methods. Amongst others, Cobb^18^ and Higuchi et al.^19^, for example, determined REE contents by NAA in the W-1 diabase RM that are very similar (within common uncertainties) to the results of more recent studies that used ICP-MS (e.g.^20^). In the case of isotope dilution analysis, for example, Hooker et al.^16^ and Langmuir et al.^21^ independently determined REE contents for the BCR-1 basalt RM that were later confirmed by ICP-MS measurements (e.g.^22^).

Despite their high analytical quality, ID and NAA data sets are usually ignored in current studies. Only complete REE data provide maximum information and allow for REE anomaly quantification. Rare earth element anomalies are one of the strongest proxies in REE research. Rare earth element anomaly quantification is based on the respective REE neighbours, which is why incomplete REE data sets are usually not suitable for this purpose. For example, missing gadolinium (Gd) poses a problem for traditional Eu anomaly quantification. Below, we solve this specific example of missing Gd. Not only ID and NAA but also modern ICP-MS data can lack certain REEs. This might be due to measurements close to quantification limits or the usage of REE spikes like Tm in REE preconcentration protocols (e.g.^23–25^). In addition, certain REE isotopes can be affected by isobaric interferences that, may not be resolved even by high-resolution ICP-MS machines, especially in high-concentration samples (e.g.^20,26^). Nevertheless, all these incomplete REE data still bear great information potential. Especially, since samples from such publications might no longer be available for re-measuring. Moreover, in other cases, sample locations might not be accessible anymore, or these samples represent past states of the sampled ecosystem (e.g., soil or water samples). Therefore, the published data sets often are the only information available and imputing the missing REEs and extracting as much information as possible from these data sets is of great scientific value and in the interest of many geoscientific sub-disciplines. Retrieving maximum information from historical measurements aligns with the current general efforts in science to make long-tail data more usable and accessible. Therefore, the purpose of this study is to demonstrate how to make use of incomplete REE data by advanced imputation methods. The methods presented here open up new possibilities for using existing high-quality REE data for new research. Furthermore, we provide the ready-to-use software tool that conducts the modelling.

This study follows O’Neill^27^ and employs polynomial modelling of logarithmic REE data to impute missing REE measurements. The polynomials relate atom size to the measured normalized contents . In the following, we refer to this approach as λ polynomial modelling (λPM) and show that it is suitable for imputing missing REE data. In addition to λPM, we apply standard polynomial modelling (SPM) with monomials as basis functions. The modelled REE data can then be used to accurately determine anomalies and interpret complete REE patterns. This initial study focuses on mafic and ultramafic rock samples. However, with rock-type adaptations and after careful testing, the method presented here should also apply to other sample types.

We conducted Monte Carlo simulations to assess the uncertainties of modelled REE data and REE anomaly quantifications. Currently, Monte Carlo simulations are not very common in geochemical data assessment. However, our results show that they have great potential to improve the interoperability and reusability of geochemical data, following the FAIR Data Principles^28^.

## Methods and data

*All data and the Python scripts used for this work can be found on Zenodo (*10.5281/zenodo.11084980).

Modern ICP-MS measurements allow the accurate and precise determination of contents for all naturally occurring REE with generally low relative standard deviations (RSD) between 3% and 15% (e.g.^29–32^). The modelled results in this study will be evaluated based on this RSD range.

### λ polynomial modelling of REE data

The basic concepts were developed by O’Neill^27^ who investigated the quantification of chondrite-normalised (sub-script CN; REE_CN_) REE patterns by polynomial modelling and the application of “*lambda shape coefficients*” (λ-parameters) for the interpretation of igneous processes. More specifically, the basis functions chosen by O’Neill^27^ are mutually orthogonal polynomials if all REEs are present in a data set, and the expansion coefficients are termed *λ shape coefficients* or λ-parameters. These λ-parameters are determined by least-squares fitting of orthogonal polynomials to match the REE pattern^27^. Although up to 14 polynomials, and hence λ-parameters, could be determined for a full REE pattern, using more than five becomes statistically insignificant^27^. In the case of basalts, O’Neill^27^ showed that for most samples, three polynomials, i.e., λ0, λ1, and λ2, are sufficient to precisely quantify their REE_N_ pattern. These first three λ-parameters also have immediate implications for the REE_N_ pattern: λ0 gives the normalised average REE content of the sample. λ1 gives the REE_N_ pattern’s slope indicating enrichment or depletion of the light vs. heavy REEs, and λ2 refers to the quadratic curvature indicating enrichment or depletion of the medium REEs.

Already in his study, O’Neill^27^ discussed that determining λ-parameters does not require complete REE patterns. The study presented here uses this principle and examines it in detail.

The λ-parameter fitting method developed by O’Neill^27^ was expanded by Anenburg and Williams^33^, who also implemented the calculations in their Python package pyrolite^34^; https://pyrolite.readthedocs.io/). For in-depth explanations of the nature and determination of these λ-parameters, the reader is referred to the two publications mentioned above as well as Anenburg^35^ and the references therein. Computations were run via Python (version 3.11.5) utilising the pyrolite package (version 0.3.5) of Anenburg and Williams^33^.

Some data in the original publications were provided in ppb or ppt. Therefore, if needed, all input data was converted into ppm (mg/kg) for easier handling.

Afterwards, λ-parameters were determined for each sample following the approach of Anenburg and Williams^33^. These determined λ-parameters constitute the coefficients in the polynomial expansion used for reconstructing the REE composition, i.e., each λ-parameter is multiplied with the corresponding orthogonal polynomial. With this λ polynomial modelling (λPM), missing REE data (e.g., ID and NAA data sets) can be imputed and used for further interpretations. Anomalously high Ce and Eu were excluded from the modelling. The anomaly detection and exclusion are included in the provided Python scripts. If, in a first modelling loop, the modelled Ce or Eu content deviates more than a set threshold (10% by default), they are marked as anomalies and excluded in the following modelling loop.

### Standard polynomial modelling of REE data

Additionally, modelling was conducted via polynomial fitting utilising the NumPy Polynomials package. The polynomial modelling was conducted similarly to the pyrolite modelling in a way that a polynomial of a given degree was fitted to the distribution of normalised REE content (y-axis) against the respective ionic radii (x-axis). In contrast to λPM , the polynomials are not orthogonal and not pre-determined. They are computed individually for each sample. Anomalous REEs were excluded from the fitting. Subsequently, the received polynomial parameters were used to re-model REE composition. A maximum polynomial degree of three was found to be best for computation (see below). The results of λPM and standard polynomial modelling (SPM) are compared and discussed below.

### Monte Carlo simulation to assess modelling precision

We conducted Monte Carlo simulations (MCSs) on λPM and SPM data from the ID-modified method verification data set to assess the uncertainties of modelled REE data. Monte Carlo simulation is a stochastic method to assess prediction uncertainties based on the input variables and a suitable probability distribution^36^. As it is unknown whether the measured data are correct or contain biases, the measured values are altered by a simulated bias using a probability distribution. For each of these artificially constructed samples, modelled REE data is computed. The result of the MCS yields a mean value for each REE element together with a bias spread in the distribution. The law of large numbers is used to get a statistically estimated measurement from the biased measurement. Due to its robustness, MCS is widely used in industry and science (e.g.^37^). In a MCS, the probable outcomes of the input model are tested repetitively, yielding a probability distribution under the given constraints. Under the assumption that the REE data errors follow a lognormal distribution, the MCSs reproduce relative uncertainties for these REE data.

As a setup for the MCSs, a normal distribution with a standard deviation of 0.1 was chosen to match the average analytical uncertainties of modern ICP-MS systems during routine operations. For each sample, 100 repetitions were computed. A comparative run with 10,000 repetitions yielded slightly different results. However, the computations of 10,000 repetitions take disproportionately longer, and 100 repetitions were sufficient for a robust assessment of the model predictions and parameter uncertainties. The results of this extensive MCS run are not included here, but the reader is referred to the Python scripts provided to re-run the MCS with individual parameters.

### Input data and data preparation

Two types of data were used: (i) complete REE data sets for method verification and (ii) isotope dilution (ID) and neutron activation analysis (NAA) data sets, that both are by nature incomplete to demonstrate the application. Method verification REE data were selected accordingly to match the sample types of the incomplete REE data. In this first study, we focused on REE data for ultramafic and mafic rocks, which are readily available.

The modelling process was verified by applying the new modelling approach to complete REE data sets from which certain REEs had been intentionally removed, allowing comparison between the modelled results and the values actually measured. The quality of the modelling method is assessed by statistical evaluation of the deviations. In this study, method verification was achieved with a data set of 14,321 mafic and ultramafic rock samples from the PetDB database (Supplementary Tab. S1;^38^; downloaded on 22nd January 2024, using the following parameters: rock type = *mafic*, *ultramafic* and *full REE data* available). The references of each publication included in this bulk data set can be found in the Supplementary Tab. S5. The downloaded PetDB data set was screened for outlier and irregular data, excluding 1,055 samples and leaving 13,266 samples to be used for the computations. Outliers are unexpected REE anomalies that, unlike the Ce and Eu anomalies, cannot be explained by natural processes. Samples with zigzag REE patterns are considered irregular data and are excluded as they usually indicate analytical issues (e.g.^30^). Since this study focuses on the development and validation of the polynomial modelling method, screened data were used.

The PetDB data set was modified in two ways: (i) praseodymium (Pr), terbium (Tb), holmium (Ho) and thulium (Tm) were removed to imitate ID data, and (ii) praseodymium, dysprosium (Dy), holmium, and erbium (Er) were removed to imitate NAA data. In the case of ID-modified data, sequential removal of Pr, Tb, Ho and Tm was also conducted to investigate the behaviour of each REE upon stepwise removal of individual REEs. The modified data sets were processed as described above. Subsequently, the modelled REE data was compared to the measured REE data, with emphasis on the removed REE.

### Terminology

In the following measured data is compared with modelled data. To avoid confusion, a certain terminology is used: For simplicity, we use the term REE “*content*” or “*data*” instead of “concentration” (content in liquid samples) or “mass fraction” (content in solid samples). The originally measured REE content is called “*measured*”, while the re-modelled REE content is ascribed as “*modelled*”. The REEs comprise 15 elements, and one measurement for one individual REE is named “*data point*”. For example, a complete REE pattern comprises 14 data points because Pm is always missing. “*Deviation*” describes the relative difference between the modelled and the measured data according to Eq. (1). In all presented REE pattern plots, the REEs are normalised to C1 Chondrite (C1) according to^39^.

1 $$\:Deviation\:\left[\%\right]=\left(\frac{modelled\:REE}{measured\:REE}-1\right)\:\times\:\:100$$

## Results and discussion

### Results of method verification – λ polynomial modelling

**Fig. 1:**
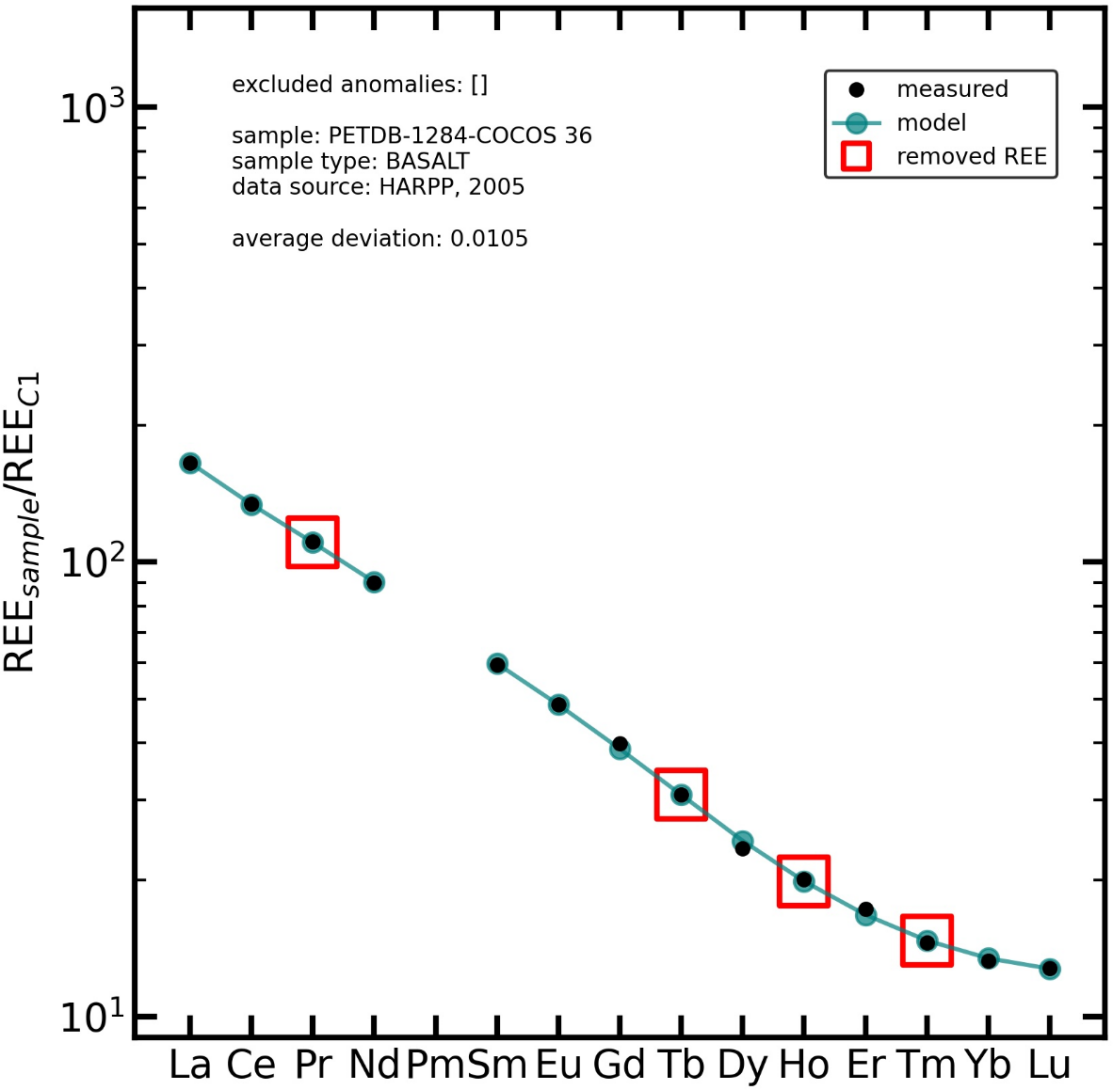
Measured and modelled REE distribution in the *PETDB-1284-COCOS 36* basalt sample. The “measured” data is from Harpp et al.^40^. Red squares indicate REEs that were removed before modelling to simulate ID datasets. The modelled REE deviate on average <2% (*average deviation: 0.0105*) from the measured data. No anomalies were excluded during the modelling, as indicated by the empty brackets.

Verification of the here developed method is best ensured by testing with complete REE data sets of the same sample type as the target data sets. Therefore, data of mafic and ultramafic rocks were used for method verification. Besides the intentionally removed REE (mimicking ID- and NAA-determined data sets), cerium (Ce) and europium (Eu) were excluded for samples showing Ce or Eu anomalies. As an example, Fig. 1 shows the *PETDB-1284-COCOS 36* basalt sample from^40^. In this case, Pr, Tb, Ho and Tm were removed prior to modelling to simulate an ID data set. The results shown in Fig. 1 demonstrate that all modelled REE data coincide with the measured data. The average deviation of the modelled REE from the measured REE is less than 2% (“average deviation: 0.0105” in Fig. 1). The largest individual deviations of modelled REE in *PETDB-1284-COCOS 36* are −3.0% for Er and 4.1% for Dy (Supplementary Tab. S2), which is within analytical uncertainty. The modelled data and the deviation for each REE in each sample can be found in the Supplementary Tab. S2 (ID modified data) and Supplementary Tab. S3 (NAA modified data).

**Fig. 2:**
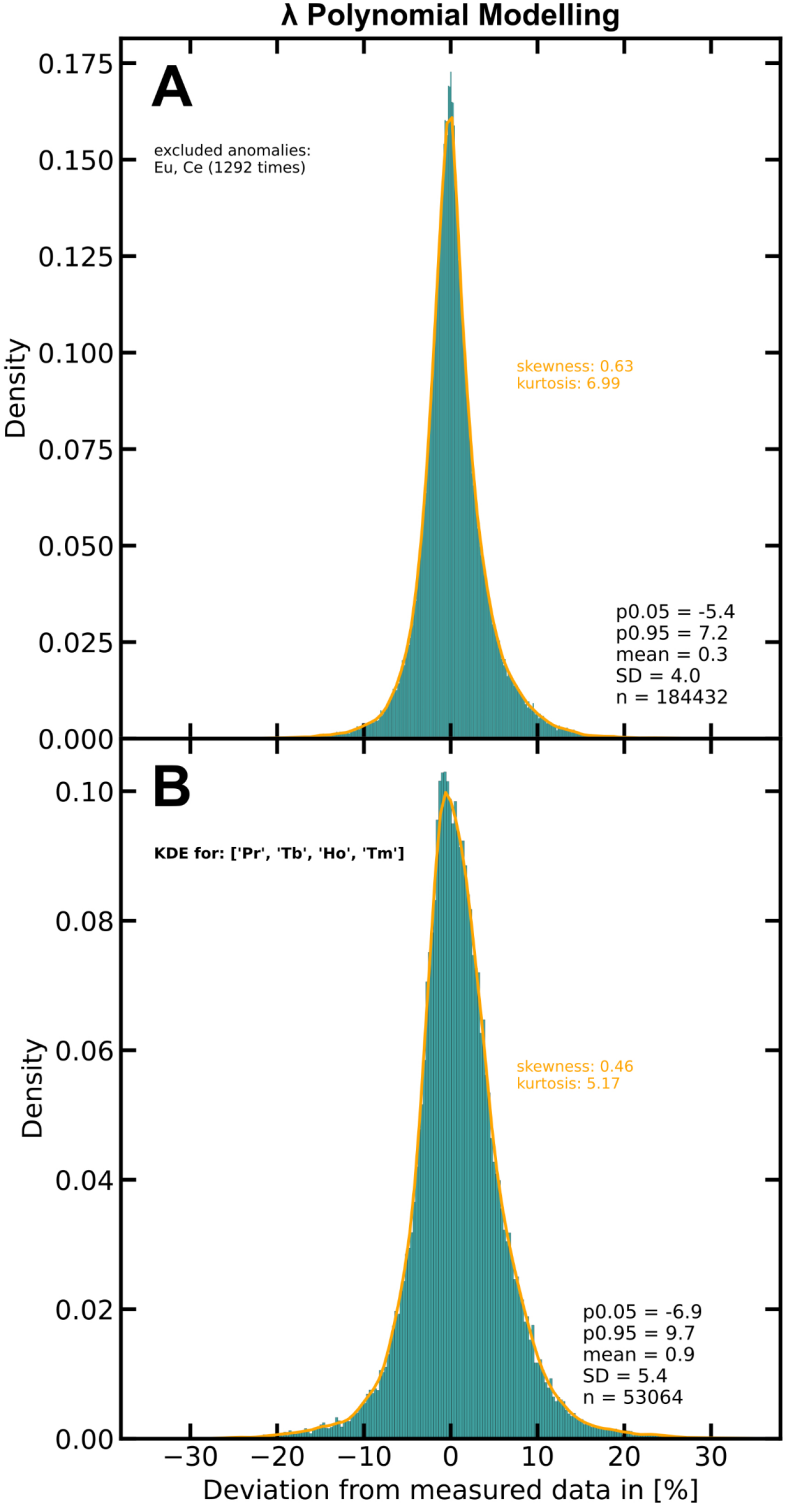
Density histograms that show the deviations of modelled REE from measured REE (ID-modified) in mafic and ultramafic rock samples from the screened PetDB dataset (https://search.earthchem.org/). The histogram data is normalised to densities. The orange lines give the kernel density estimation curves. Cerium and Eu were excluded 1,292 times as anomalies during modelling. (**A**) The histogram for all REEs shows a skewness of 0.63, a kurtosis of 6.99. 90% and the data points range between −5.4% and 7.2% (5%–95% percentile; SD = 4). (**B**) The histogram for the removed Pr, Tb, Ho and Tm shows a skewness of 0.46, a kurtosis of 5.17. 90% and the data points range between −6.9% and 9.7% (5%–95% percentile; SD = 5.4).

A summary of all deviations between modelled REE and measured REE in the method verification data set (ID modified) is shown in the density histogram of (Fig. 2A). The x-axis shows the deviation between modelled and measured REEs, with values close to zero indicating a high accuracy of the modelling process. The method verification data set contains 184,432 individual data points, and Ce and Eu were excluded 1,292 times. The deviations are narrowly distributed around a mean of 0.3%. Almost all data points show deviations of less than 10% (absolute value), with 90% of the data points ranging between − 5.4% and 7.2% (5% and 95% percentile; SD = 4.0). The kernel density estimation (KDE) curve is almost perfectly symmetric with a skewness of 0.63. A perfectly symmetric distribution, like the normal distribution, has a skewness of zero. However, skewness values between − 1 and 1 indicate only minor asymmetry. The high symmetry of the KDE and the mean close to zero prove that the applied method does not introduce bias to the modelled REE contents. In this case, the KDE’s kurtosis (normalised fourth central moment) of 6.99 indicates that outliers are very minor, corroborating the high precision of the applied method. For comparison, a normal distribution has a kurtosis of 3, thus the value of 6.99 indicates a narrower distribution than the normal distribution.

Figure 2B shows the deviations between modelled and measured data for Pr, Tb, Ho, and Tm, i.e., for those REE that had been intentionally removed. They show slightly larger deviations than the remaining REEs but still are also mostly <10% in a narrow range between − 6.9% and 9.7% (5% and 95% percentile; SD = 5.4).

Figure S1 (A–C; Supplementary) shows how the deviation between modelled data and measured data of each REE changes upon the sequential removal of Pr, Tb, Ho and Tm. The p0.05, p0.95, mean, standard deviation, skewness and kurtosis for each KDE curve can be found in the Supplementary Tab. S6. The solid black KDE curve refers to REE modelling with the complete verification data set. These baseline KDEs cover narrow ranges for each REE and are predominantly symmetrical around zero, with some minor exceptions. The blue, orange, green and red KDE curves refer to the modelled data after the sequential removal of Pr, Tb, Ho and Tm. For all REEs that are not removed (La, Ce, Nd, Sm, Eu, Gd, Dy, Er, Yb, and Ho) from the input data set, the different KDE curves are almost identical, indicating that the removal of other REEs does not affect their modelling. The only exceptions are Er and Yb, which show slightly thinner KDE curves after Tm removal. Neither case indicates a loss of quality for modelling. The removal of Pr, Tb, Ho and Tm from the input data set affects their respective modelling. After removal, the slightly broader KDE curves of each element indicate larger deviations between modelled and measured data. However, most modelled REE data still has deviations less than 10% (absolute value; Supplementary Tab. S6). Furthermore, the KDE curves for Pr, Tb, Ho and Tm only change after the respective REE is removed from the input data set and are not affected by the removal of further REEs: The KDE for Tb after Pr removal is identical to that of Tb baseline KDE; the Ho KDE curves after Pr removal and after Pr and Tb removal are identical to the Ho baseline KDE; the Tm KDE curves after Pr, Tb and Ho removal are similar to that of Tm baseline KDE. This behaviour shows that the λPM is mostly independent from neighbouring REEs. Mathematically, this result is expected as the REE patterns are smooth and the remaining fittings points for computing the regression are sufficient. Removing La or Lu might have more impact than removing in-between values. The application of λPM is not limited to ID and NAA data sets, as presented here. However, although there surely is a limit of how many REEs can be removed from a data set before the modelling yields erroneous results, this limit was not reached for the ID- or NAA-modified data sets presented above. We did not conduct extensive testing on when the modelling fails. Our experience is that, when distributed equally, at least half of the REEs can be removed. If neighbouring REEs are removed, the breaking point might be around four or five, depending on the sample and what is considered an erroneous result. However, the modelling is most sensitive to removing REEs at the edges. For example, removing just La and Ce might already lead to erroneous results for the remaining light REEs.

Like the above-described computations on the ID-modified data set, method verification was also conducted on an NAA-modified version of the PetDB data set. Modelling of the NAA-modified data yields results almost identical to the ID-modified modelling. Figure S2a shows the KDE plot for the deviations of modelled REE and measured REE in the NAA-modified data set. The deviations cluster in a narrow range around a mean of −0.6% with 5% and 95% percentiles of −7.3% and 6.0%, respectively (SD = 4.3, kurtosis = 5.84). Figure S2b shows the KDE plot for the deviations of Pr, Dy, Ho and Er (NAA-modification). This KDE is slightly shifted to a mean of −2.2, indicating a minor underestimation of the removed REE by modelling. A closer look at the individual deviations (Fig. S3) shows that while the deviation KDE for Pr is centred at almost zero (mean = −0.5), the KDEs for Dy, Ho and Er are shifted towards the left side with decreasing means between −2.4 and −3.3. Mathematically, the explanation for this behaviour is that Dy, Ho and Er are direct neighbours, creating a rather large gap of three missing values between Tb and Tm. In comparison, for the ID-modified data, each removed REE was surrounded by two fixed REE data points that supported the fitting. Nevertheless, the modelled data for Dy, Ho and Er in the NAA-modified data set predominantly show deviations <10% and are, therefore, in good agreement with typical uncertainties of modern routine analytical methods for REEs.

Overall, the low deviations between modelled and measured data and the high reproducibility, even after removing multiple REEs, prove the accuracy and precision of the applied modelling approach. The large number of data points in the method verification data set also demonstrates its applicability to a wide range of mafic and ultramafic lithologies with a range of different REE compositions. The deviations between modelled and measured data fall well-within typical analytical uncertainties for REE measurements by ICP-MS. Therefore, the λPM method is suitable for imputing incomplete REE data sets of mafic and ultramafic rocks.

### Optimal polynomial degree for standard polynomial modelling

**Fig. 3:**
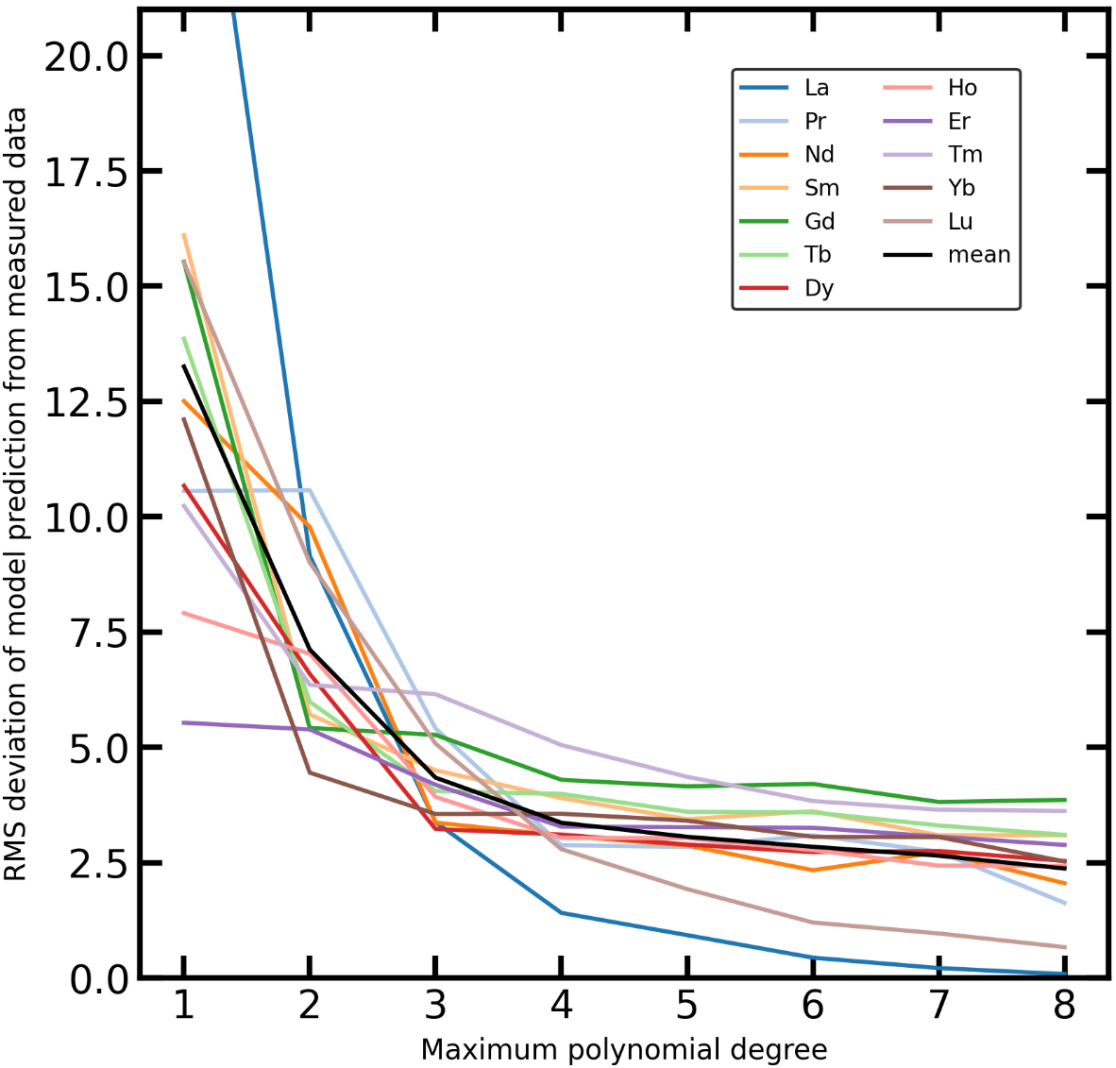
Root mean squared (RMS) deviation of model prediction from measured data in PetDB dataset. SPM was conducted with maximum polynomial degrees from one to eight. Cerium and Eu are not shown, since they were excluded in many samples’ fitting due to anomalies. The RMS deviation of each REE shows asymptotic behaviour, approaching a lower limit of around 5% at a maximum polynomial degree of three. This behaviour indicates that a maximum polynomial degree of three is optimal, while higher degrees can cause overfitting.

Fitting a polynomial to a data set as a regression model for estimating missing values always bears the risk of overfitting. O’Neill^27^ showed that for the λ-parameter fitting, the maximum polynomial degree should be four, while for most samples, even degree three is sufficient. To determine the optimal maximum polynomial degree for the standard polynomial modelling (SPM), the entire PetDB data set (no REEs removed) was computed for polynomials of degrees one to eight, yielding eight sets of modelled REE data. For comparison, the root mean square (RMS) deviation of model prediction from measured values for each REE was computed in each of the eight model data sets. The RMS deviation was used to ensure positive values, and the results are shown in (Fig. 3). Cerium and Eu are not shown due to their anomalous high or low content in many samples, resulting from their specific redox-sensitivity. The RMS deviations of the REEs show asymptotic behaviour, approaching a lower limit of around 5% at a polynomial degree of three. This behaviour shows that increasing polynomial degrees from one to three also increases the prediction quality. Almost constant RMS deviations above degree three indicate overfitting when polynomials of degree four and higher are used. Only the RMS deviations of La and Lu continue to decrease slightly above degree three. However, this is due to the position of La and Lu at the very beginning and end, respectively, of the REE patterns. Due to the nature of polynomial modelling, data at the edges are more strongly affected by the order of the polynomial. Therefore, we suggest that a maximum polynomial degree of three be chosen for SPM to model REE data. This is consistent with the observation of O’Neill^27^, even if he decided to choose four. The RMS deviations of the REEs at a polynomial degree of three range from approximately 3%–7%, with a mean close to 5%.

### Results for standard polynomial modelling

The standard polynomial modelling was applied to the ID-modified PetDB data set. As described above, a maximum polynomial degree of three was selected for the modelling. The results can be found in the Supplementary Tab. S4 and are summarised in two histogram plots (Fig. S4) that show the deviation between the modelled and measured data for all REE (Fig. S4a) and exclusively for the removed Pr, Tb, Ho and Tm (Fig. S4b). These two histograms are counterparts to (Fig. 2). The SPM yields good overall modelling results, with most data points showing deviations less than 10%. In Figure S4a, 90% of the data range between −6.2% and 8.4% deviation (SD = 4.9). In Figure S4b, which only shows the deviations for the removed REE, 90% of the data points range between −6.7 and 11.1% (SD = 6.4). Thus, the SPM yields slightly higher deviations compared to the λPM. However, it should be noted that the SPM was run with a polynomial degree of three, while the λPM was run with a maximum polynomial degree of four. The results of SPM are similar to those of λPM since both methods share the same principle of fitting the REE distribution pattern to polynomials.

### Results for Monte Carlo simulation of modelled REE data from ID-modified PetDB data set

**Fig. 4:**
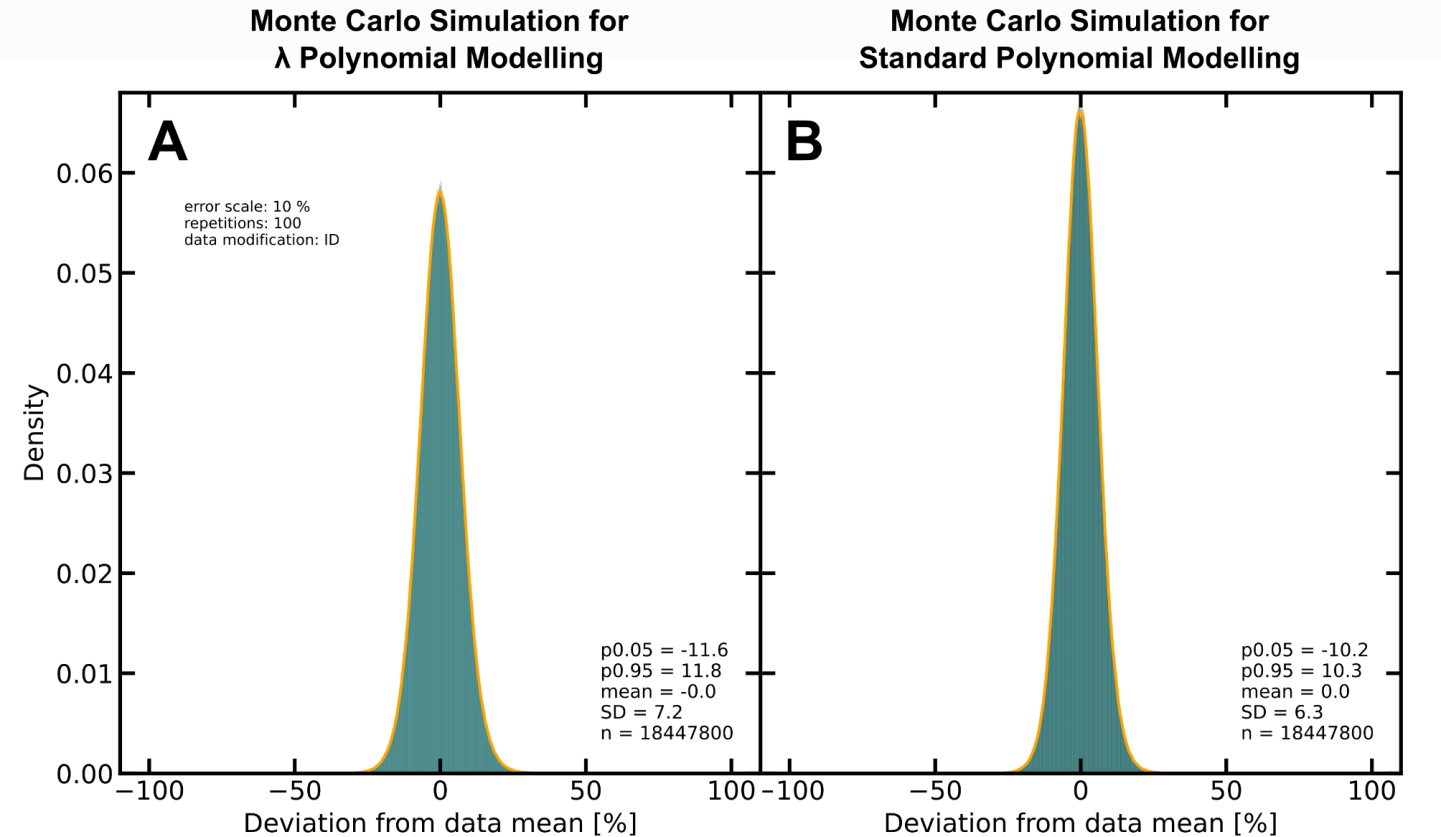
Density histograms that show the deviations from the mean of Monte Carlo simulations utilising (**A**) λPM and (**B**) SPM. For both Monte Carlo simulations, 100 repetitions and a normal distribution with a standard deviation of 0.1 were chosen. The SPM shows slightly smaller deviations (SD = 6.3) than the λPM (SD = 7.2) throughout the Monte Carlo simulation.

A simple Monte Carlo simulation was run on the ID-modified PetDB data set for both modelling methods, λPM and SPM. The results are visualised in Fig. 4 by means of histograms showing the deviations from the mean of each REE in each sample throughout 100 repetitions.

The Monte Carlo simulation for SPM yields slightly smaller uncertainties of approx. ±10.3% (5%–95% percentile; SD = 6.3) than the Monte Carlo simulation of λPM with approx. ±11.8% (5%–95% percentile; SD = 7.2). However, these marginal differences may be neglected as both modelling methods yield uncertainties within the typical analytical uncertainties for REEs.

These computations show that Monte Carlo simulation is a vital tool for assessing uncertainties of modelled REE data. Especially for REE anomaly quantification, the Monte Carlo simulation is a promising approach to assess the uncertainties. Classically, REE anomalies are quantified by calculating a theoretical non-anomalous REE* content (e.g.^41,42^). Since this REE* value is not measured but only computed, uncertainties have usually not been fully assessed in the past. However, Monte Carlo simulation can now be applied to estimate REE* uncertainties robustly. An example is given further below. Holistic and accurate determinations of REE anomaly uncertainties are crucial to derive conclusions from REE anomaly quantification.

The Monte Carlo simulation might also be fine-tuned in future applications using individual probability distributions for each REE depending on their respective analytical uncertainty within a measurement. For example, one could use a sample-specific probability distribution based on large data sets of the same sample type.

### Final comparison λ polynomial and standard polynomial modelling

The modelling results obtained with the λPM and SPM are very similar. While the Monte Carlo simulation yields slightly lower uncertainties for SPM than for λPM, the fitting accuracy is higher for the latter. An example is given in Supplementary Figure S5, showing the measured REE in comparison to the λPM (Fig. S5a) and SPM (Fig. S5b) data. By simple visual inspection, it is obvious that the λPM yields the more accurate and realistic REE model. In the following, we will use the λPM for re-modelling REE data from originally incomplete REE data sets.

### Results for imputing incomplete REE data

**Fig. 5:**
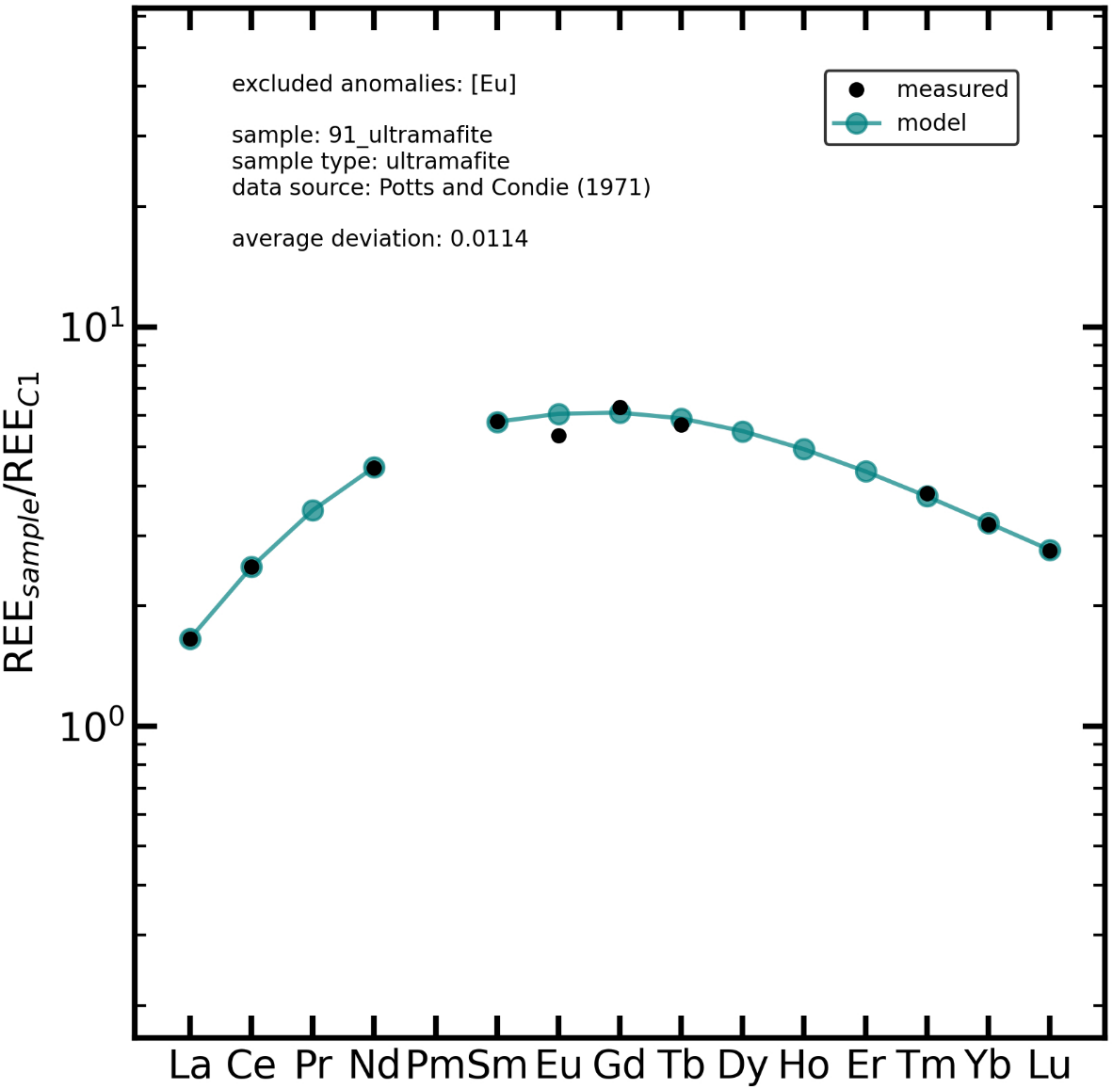
Measured and modelled REE distribution in the *91_ultramafite* ultramafite sample. The “measured” data is from Potts and Condie^43^ and was determined by NAA. Praseodymium, Dy, Ho and Er were not measured but modelled with the here presented method. Europium was excluded as an anomaly during modelling. The remaining modelled REE (La, Ce, Nd, Sm, Gd, Tb, Tm, Yb, and Lu) deviate on average <2% (*average deviation: 0.0114*) from the measured data.

All results for the modelled data of originally incomplete REE data sets can be found in (Supplementary Table 7). Figure 5 shows an example from the NAA data set of Potts and Condie^43^ (*91_ultramafite* is an ultramafic rock sample). Data for Pr, Dy, Ho, and Er are missing in the data set^43^ because NAA was used. Additionally, Eu was excluded during the modelling process as a slight negative Eu anomaly was detected. The modelled data coincides with the originally measured data for the remaining REEs with an average deviation of less than 2%. Gadolinium (−3.0%) and Tb (3.7%) show the largest deviations, which probably originate from the fact that Gd and Tb are surrounded by Eu (left side) and Dy, Ho, Er (right side), which were all either excluded during modelling or missing in the input data set. Nevertheless, deviations of <4% (absolute value) are remarkably good and much lower than typical relative standard deviations for the analysis of REE. Further modelled REE data and deviations for each sample from Potts and Condie^43^, Zindler et al.^44^, and Stosch et al.^45^ can be found in (Supplementary Table 7).

**Fig. 6:**
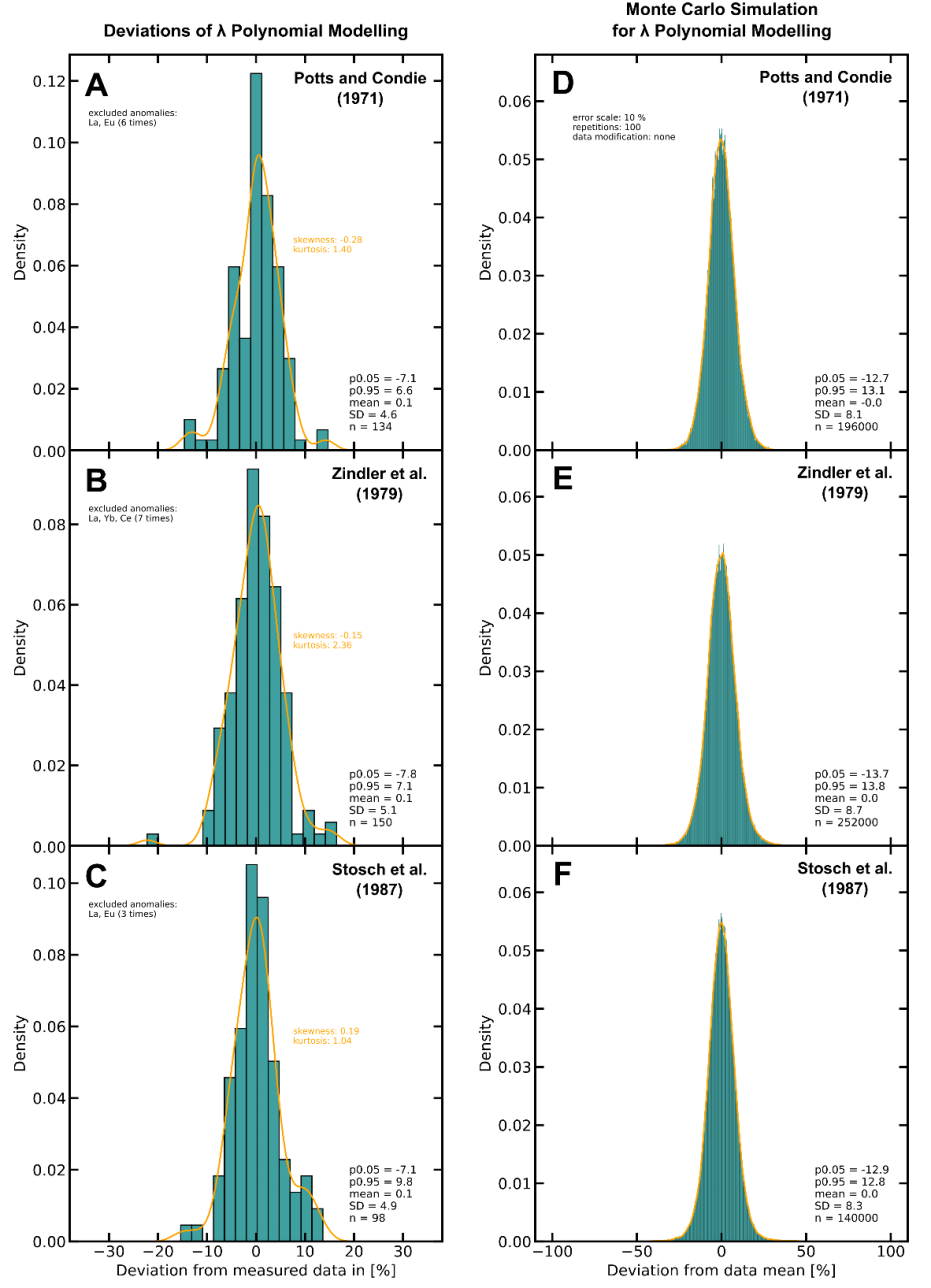
Left side (**A**–**C**) histograms show deviations of modelled REE (λPM) from measured REE. Right side (**D**–**F**) histograms show deviations of Monte Carlo simulation data from the respective mean. The orange lines give the kernel density estimation curves. All KDEs show considerably small skewnesses. Excluded anomalies are given in the respective upper left corners. (**A**) Ultramafic rock and clinopyroxene samples from Potts and Condie^43^, with 90% of the data points ranging between −7.1% and 6.6% (5% and 95% percentile; SD = 4.6). (**B**) Basalt and tholeiite samples from Zindler et al.^44^, with 90% of the data points ranging between −7.8% and 7.1% (SD = 5.1). (**C**) Ultramafic rock, pyroxene and garnet samples from Stosch et al.^45^, with 90% of the data points ranging between −7.1% and 9.8% (SD = 4.9). Monte Carlo simulation input data from (**D**) Potts and Condie^43^, (**E**) Zindler et al.^44^ and (**F**) Stosch et al.^45^. Monte Carlo simulations were conducted with 1000 repetitions, and a normal distribution with a standard deviation of 0.1 were chosen. The MCS for all three datasets yield estimated uncertainties around ± 14% with standard deviations of 8.1 (**D**), 8.7 (**E**) and 8.3 (**F**).

A summary of the deviations between modelled and measured REE of^43–45^ for existing REE data in the input data sets can be found in Fig. 6a-c, respectively. All three data sets show means close to zero (all 0.1%) and small skewnesses (−0.28 to 0.19). The majority of modelled data shows deviations less than 10%, with 5% and 95% percentiles of −7.1 and 6.6% (SD = 4.6;^43^), −7.8% and 7.1% (SD = 5.1;^44^), and −7.1% and 9.8% (SD = 4.9;^45^), respectively. Detailed deviations for each REE can be found in Supplementary Table 7, which also contains data from three olivine^43^, two dunite^46^, two ferrohedenbergite, and two fayalite samples from^47^.

### Uncertainties of modelled REE data assessed by Monte Carlo simulation

Monte Carlo simulations for λPM data were conducted for each of the three incomplete REE data sets, as described above. The only difference is that 1,000 repetitions were conducted during the MCS for the three originally incomplete data sets. Summaries of the Monte Carlo simulations are given in Fig. 6d–f, showing the histograms for the deviations from the data mean. All three data sets show similar distributions with standard deviations of 8.1^43^, 8.7^44^ and 8.3^45^. These uncertainties are somewhat larger than those determined via MCS for the ID-modified PetDB data set (Fig. 4a) and also larger than the plain deviations determined in (Fig. 2). Therefore, the 5% and 95% percentiles given in Fig. 6d–f can be considered rather conservative uncertainties. Nevertheless, these determined uncertainties range within common analytical uncertainties for REEs.

Regardless of the sample type, modelled REE data shows remarkably good agreement with the measured REE data for available REEs. Based on these low deviations and the above results for the method verification and MCS, it can be assumed that the modelled REE contents of missing REEs are accurate within the above-given uncertainties.

### Outcome and application of imputed REE data

**Fig. 7:**
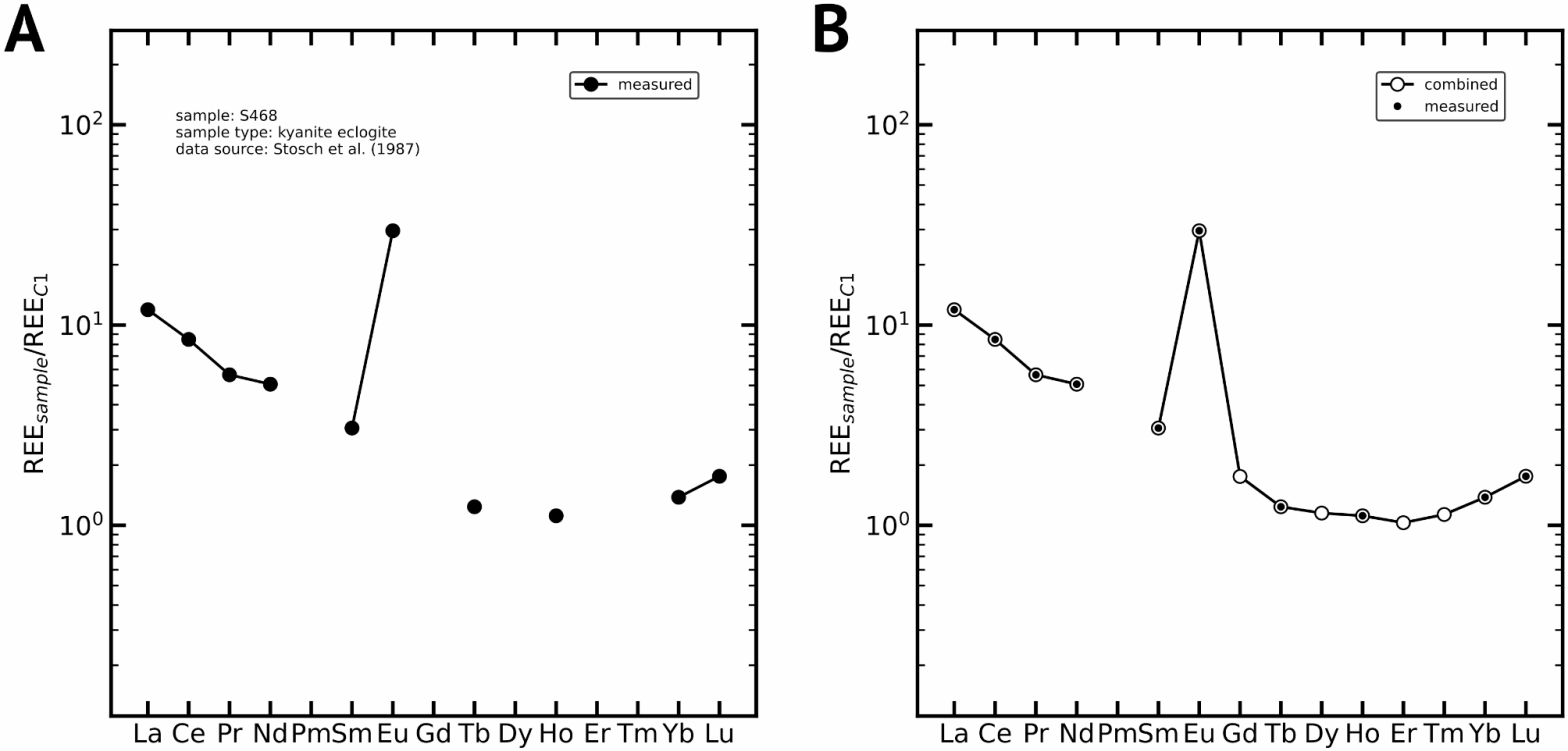
Measured and modelled REE of the *S468* kyanite eclogite sample^45^. (**A**) shows only the measured REE data. (**B**) shows the combination of measured and modelled data. Data normalised to C1 chondrite^39^. The sample shows a prominent Eu anomaly that can be quantified properly after restoring REE contents via the λparameters.

The combination of measured and modelled REE data yields full REE distribution patterns that can be evaluated like other complete data sets. Figure 7b combines the measured (Fig. 7a) and modelled data of the kyanite eclogite sample *S468* from Stosch et al.^45^. Although already prominent in the measured data set, the Eu anomaly can now be evaluated and properly quantified. Anomaly quantification can be conducted using traditional arithmetic or geometric mean determination methods. Alternatively, anomalies can also be computed using the modelled REE data^27^. For basalts, computation via λPM (*shape coefficient modelling*) improves the Eu anomaly quantification by 18%^27^. Thus, we highly recommend using the λPM method as it considers all REEs and not just two neighbouring REEs. In the case of *S468*, the C1normalised (after^39^) measured [Eu]_C1_ = 29.58 and the modelled [Eu]_C1_* = 2.25. Therefore, the Eu anomaly ([Eu]_C1_ / [Eu]_C1_*) = 13.14. For comparison, an Eu anomaly quantification via measured Sm and Tb (Eq. (2); after^48^) yields a value of 12.03 ([Eu]_C1_* = 2.46), which is 8% lower than the former.

2 $$\:{Eu}_{C1}^{*}=\:0.67\times\:{Sm}_{C1}+0.33\times\:{Tb}_{C1}$$

**Fig. 8:**
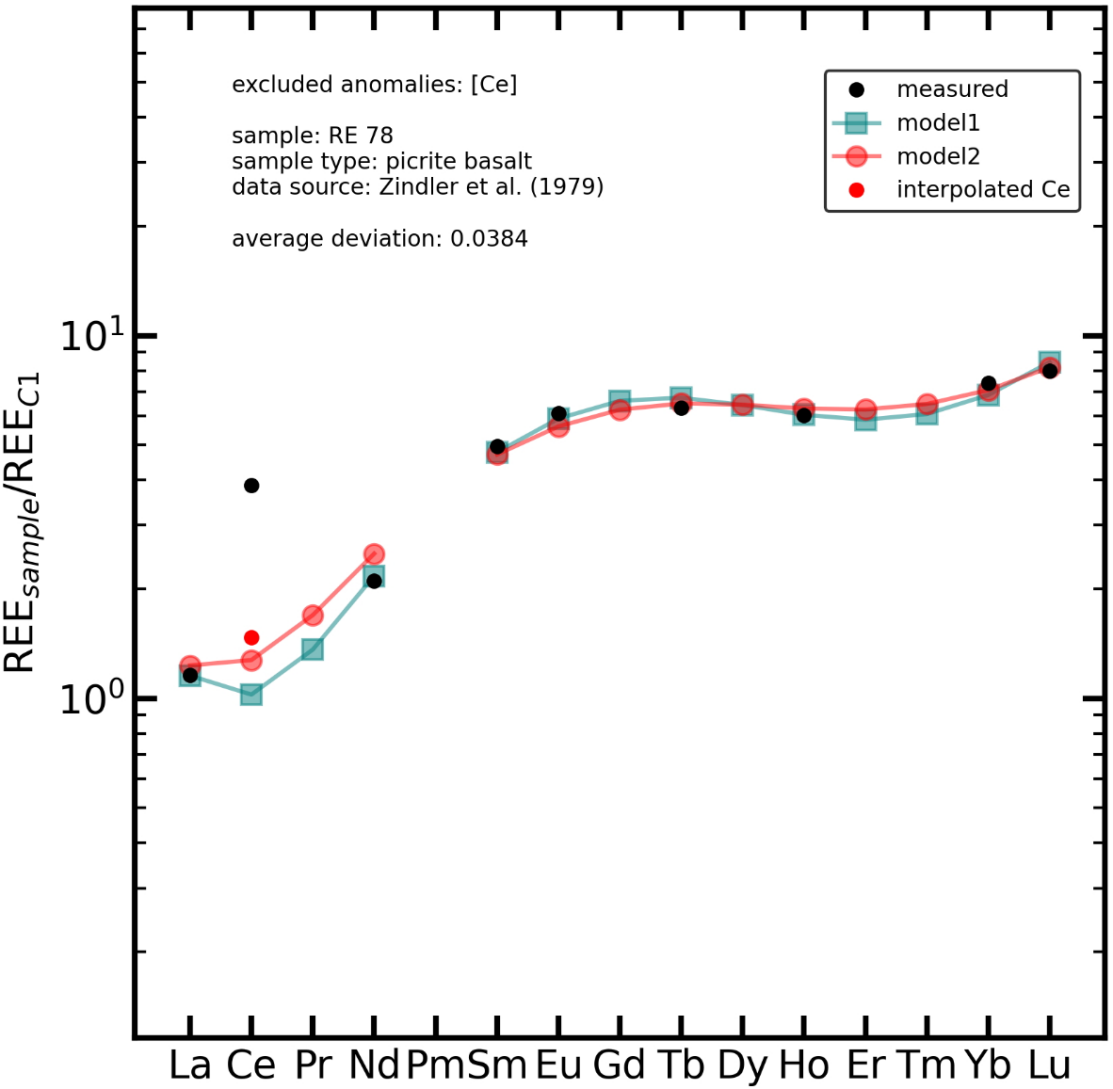
Measured and modelled REE in the *RE 78* picrite basalt sample^44^. Data normalised to C1 chondrite^39^. The sample shows a prominent Ce anomaly (black dots mark measured data). The green graph (“*model 1*”) shows the modelled REE with Ce excluded during modelling. The red dot marks the linearly interpolated Ce (Eq. 3). The red graph (“*model 2*”) shows the modelled REE data considering the linearly interpolated Ce. *Model 2* shows larger deviations from the actual measured data compared to *model 1*, especially for La and Nd. Also, *model 2* shows a significant deviation for Ce.

Another example is given in Fig. 8 for the picrite basalt sample *RE 78*^44^ showing a strong positive Ce anomaly. Therefore, Ce was excluded from the modelling (“*model 1*” in Fig. 8), yielding good agreement with the remaining measured REE. However, *model 1* has a comparably low value for Ce, causing a slight depression between measured La and Nd. This may look odd at first glance, suggesting a potential La anomaly. Therefore, for *model 2*, Ce was determined by linear interpolation between La and Nd first (Eq. 3). Afterwards, modelling was computed with the linearly interpolated Ce. However, Fig. 8 clearly shows that *model 2* has larger deviations from the measured REE, especially for La (5.6%) and Nd (17.0%), than *model 1* (La: −0.4%; Nd: 3.1%). Also, there is a significant difference between the linearly interpolated Ce (0.88 ppm), which was included in the input file for *model 2*, and the modelled Ce value of *model 2* (0.77 ppm). Considering the overall smoothness and fit, *model 1* is preferred, strongly arguing for REE anomaly quantification using λPM instead of interpolation between two neighbouring REEs, as already discussed^27^. Furthermore, this example shows that λPM might even help to uncover other hidden anomalies, like La in the *RE78* sample. This has to be examined in more detail in the future.

3 $$\:{log}_{10}\left({Ce}_{C1}^{*}\right)=\:0.67\times\:{{log}_{10}(La}_{C1})+0.33\times\:{{log}_{10}(Nd}_{C1})$$

To take the REE anomaly determination one step further, the precision of the REE anomaly quantification can be estimated using MCS. For example, the kyanite eclogite sample *S468* (Fig. 7) is used. Stosch et al.^45^ give a 2σ standard deviation of 0.04 mg/kg for *S468*, which corresponds to a single relative standard deviation of 1.15% (Eu_S468_ = 1.74 mg/kg; 1σ SD = 0.02 mg/kg). The standard deviation for Eu_S468_ in the here conducted MCS is 0.01 mg/kg, yielding a relative standard deviation for the modelled Eu_S468_* of 7.7% (Eu_S468_* = 0.13). Since REE anomaly calculation is a division, the uncertainties add up to 8.0% (Eq. 4) in the case of *S468*. This approach makes REE anomaly calculations more comparable, offering an improved way to compare REE data of different samples. This approach is not limited to the REE but can also be applied to quantifying Y anomalies in REY distributions. Generally, MCS can be applied to assess uncertainties of anomalies in spider diagrams (e.g.^49^).

4 $$\:relative\:uncertainty=\:\sqrt{{\left(2.3\:\%\right)}^{2}+{\left(7.7\:\%\right)}^{2}}=8.0\:\%$$

Another application for the presented λPM are REEs affected by interferences, which cannot be resolved analytically. Especially in high-concentration samples, like ores, many REE isotopes can be affected by isobaric interferences (e.g.^20,26^). Even the light REE can interfere on their heavy neighbours. For example, Anenburg et al.^50^ demonstrated one way of correcting Er, Gd, Tb and Yb. The here presented λPM now provides another robust and accurate method to impute interference-affected REE data. The provided scripts offer an easy application for every researcher.

## Remarks

Restoring missing REEs remains primarily a mathematical imputation procedure that involves minimal geochemical modelling since the polynomials used are based on the atomic radii of the REEs. Therefore, this method is a data approximation and should be cautiously applied, especially regarding potentially anomalous elements. A profound inspection of the modelled data is necessary. Nevertheless, the presented method here is much more elaborate than simple linear or geometrical interpolation, as it involves the entire REE data set. If applied correctly, it offers a great opportunity to reassess incomplete REE data and quantify REE distributions and anomalies. This is not only limited to ID and NAA data but can also be applied to ICP-MS measurements in which not all REEs were determined. However, especially in the case of REE measurements close to limits of quantification, the λPM or SPM do not replace a re-measurement if the sample is still available.

## Conclusions

In this study, we conducted λ polynomial and standard polynomial modelling to impute incomplete REE data sets. Both methods are capable of quantifying REE distribution patterns and modelling missing REE data well-within the uncertainties of modern ICP-MS analytical methods (<10%). The λPM is based on the initial work of O’Neill^27^ and its continuation and pyrolite implementation by Anenburg and Williams^33^. Method verification was achieved by processing REE data of >13,000 mafic and ultramafic rock samples from the PetDB database^38^. Since this study focused on imputing isotope dilution (Pr, Tb, Ho and Tm missing) and neutron activation analysis (Pr, Dy, Ho and Er missing) data, the method verification data sets were modified accordingly. Nevertheless, λPM and SPM are also applicable to data sets in which other REEs are missing. Additionally, Monte Carlo simulations were conducted to assess the precision of the model predictions, which were also found to fall well-within the analytical uncertainties of modern REE analysis, confirming the reliability of the here presented method.

The data processing methods we presented offer a new opportunity to utilise incomplete REE data. This allows to reassess not only highly valuable REE data sets, such as those measured with ID and NAA, but also REE data sets where certain REEs were used as spikes or are affected by interferences in the analysis. Furthermore, the combination of λPM and MCS is capable of precise REE anomaly quantification and determination of REE anomaly uncertainties, an important prerequisite for quantitative REE anomaly comparison. We thus recommend implementing these methods in future REE investigations to ensure objectivity and an enhanced inter-comparability of different REE studies. The methods presented here aim to make geochemical data more FAIR^28^.

## Electronic supplementary material

Below is the link to the electronic supplementary material.

Supplementary Material 1

Supplementary Material 2

## Data Availability

Data and Python scripts are available through Zenodo at https://doi.org/10.5281/zenodo.11084980. If there are any problems, accessing the data or running the scripts, please contact David Ernst (ORCiD: https://orcid.org/0000-0003-4316-135X) or Malte Mues (ORCiD: https://orcid.org/0000-0002-6291-9886).

## Declarations

### Generative AI and AI-assisted technologies in the writing process

During the preparation of this work the authors used Grammarly in order to spell check. After using this tool, the authors reviewed and edited the content as needed and take full responsibility for the content of the publication.
