# Supplementary material for: Polynomial modelling of high-quality yet incomplete rare earth element data sets and a holistic assessment of REE anomalies
Source: Sci Rep. 2025 Feb 13;15:5360. doi: 10.1038/s41598-025-89227-2 (PMC11825873; doi:10.1038/s41598-025-89227-2)
Supplement: Supplementary file 2 — Supplementary Material 2 [file 41598_2025_89227_MOESM2_ESM.pdf]

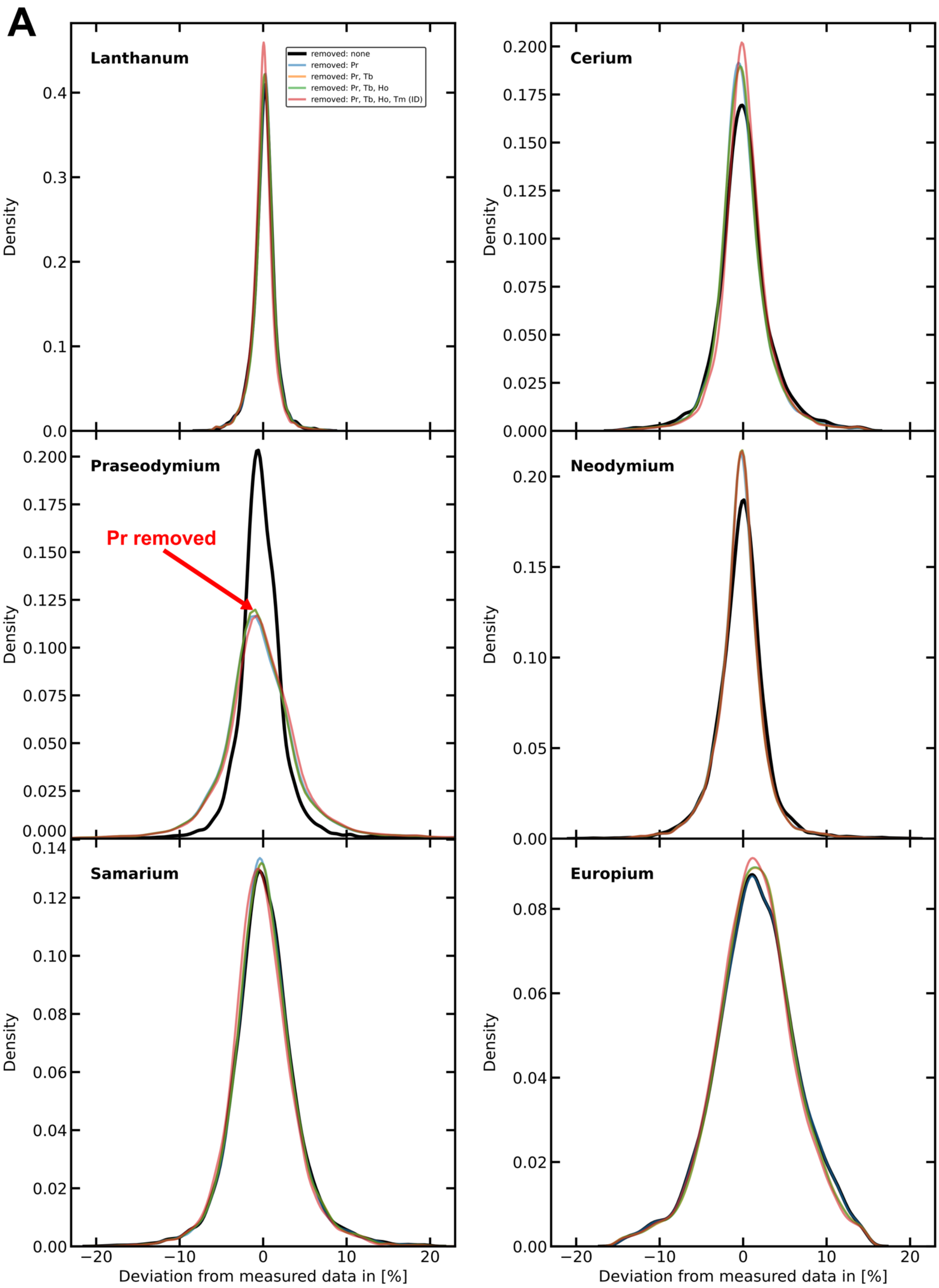

A
Lanthanum
Cerium
Praseodymium
Neodymium
Samarium
Europium
removed: none
removed: Pr
removed: Pr, Tb
removed: Pr, Tb, Ho
removed: Pr, Tb, Ho, Tm (ID)
Pr removed
Density
Deviation from measured data in [%]

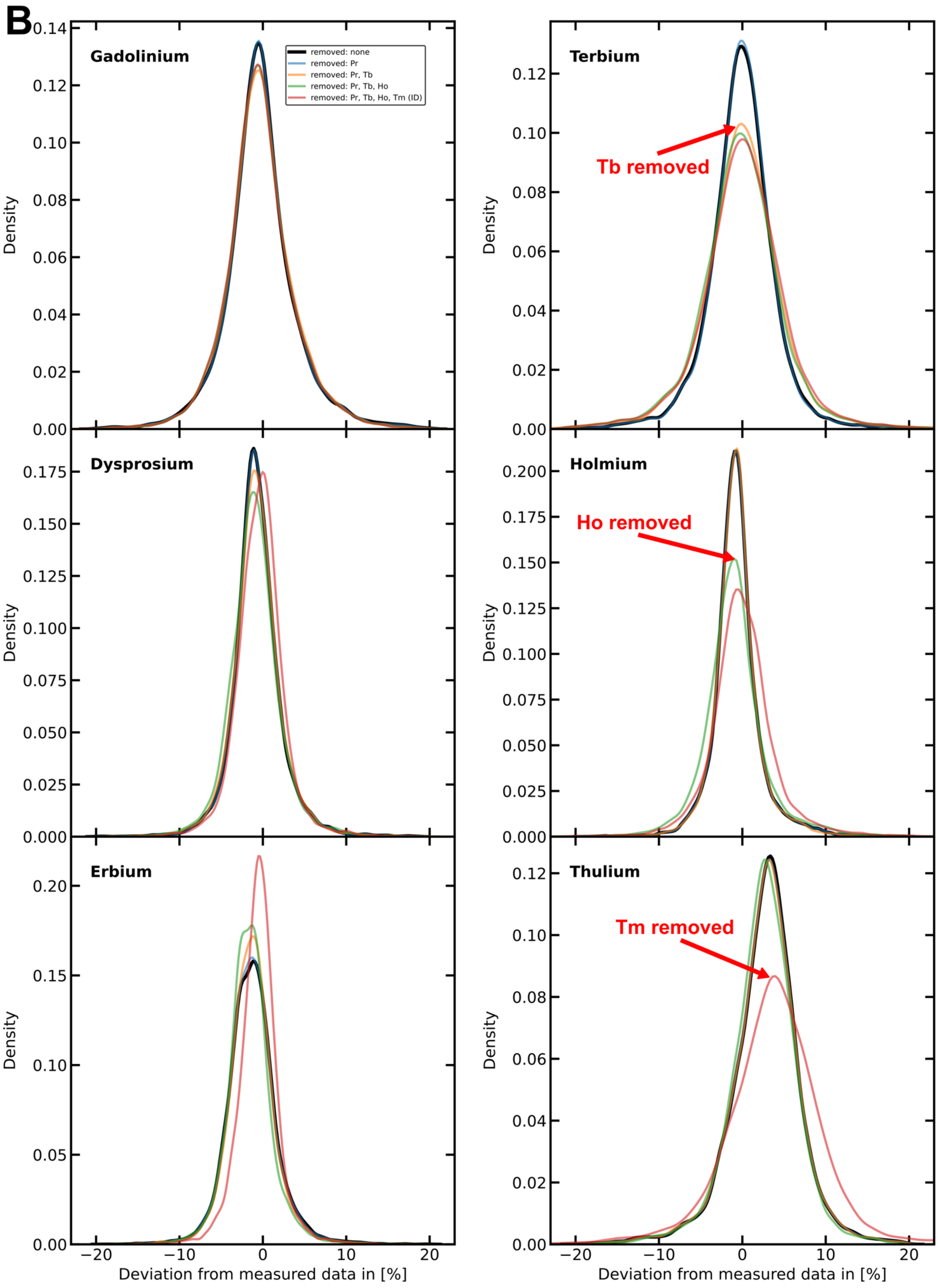
B
Gadolinium
removed: none
removed: Pr
removed: Pr, Tb
removed: Pr, Tb, Ho
removed: Pr, Tb, Ho, Tm (ID)
Terbium
Tb removed
Dysprosium
Holmium
Ho removed
Erbium
Thulium
Tm removed
Density
Deviation from measured data in [%]
0.00
0.02
0.04
0.06
0.08
0.10
0.12
0.14
0.000
0.025
0.050
0.075
0.100
0.125
0.150
0.175
0.200
0.05
0.15
0.20
−20
−10
0
10
20

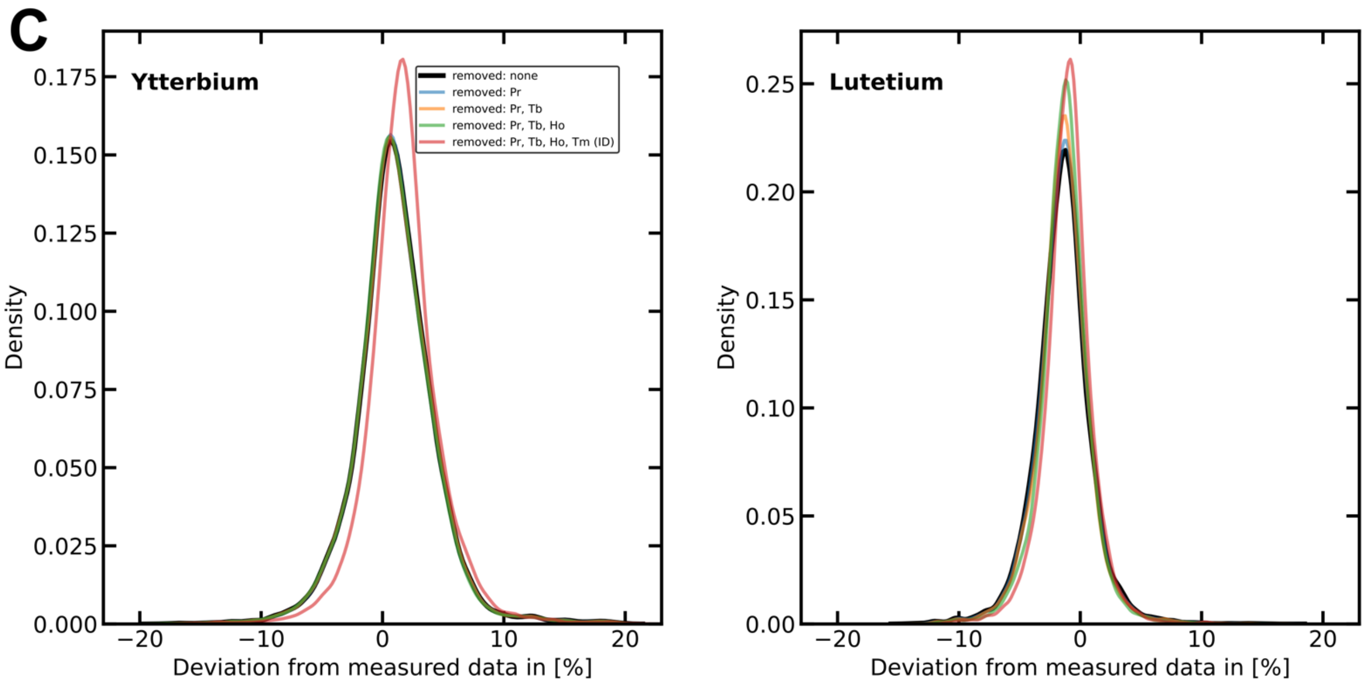

Figure S1: KDE curves for the deviations between modelled and measured data for each REE and the stepwise sequential removal of Py, Tb, Ho and Tm (ID modification). For further explanations, see the text.

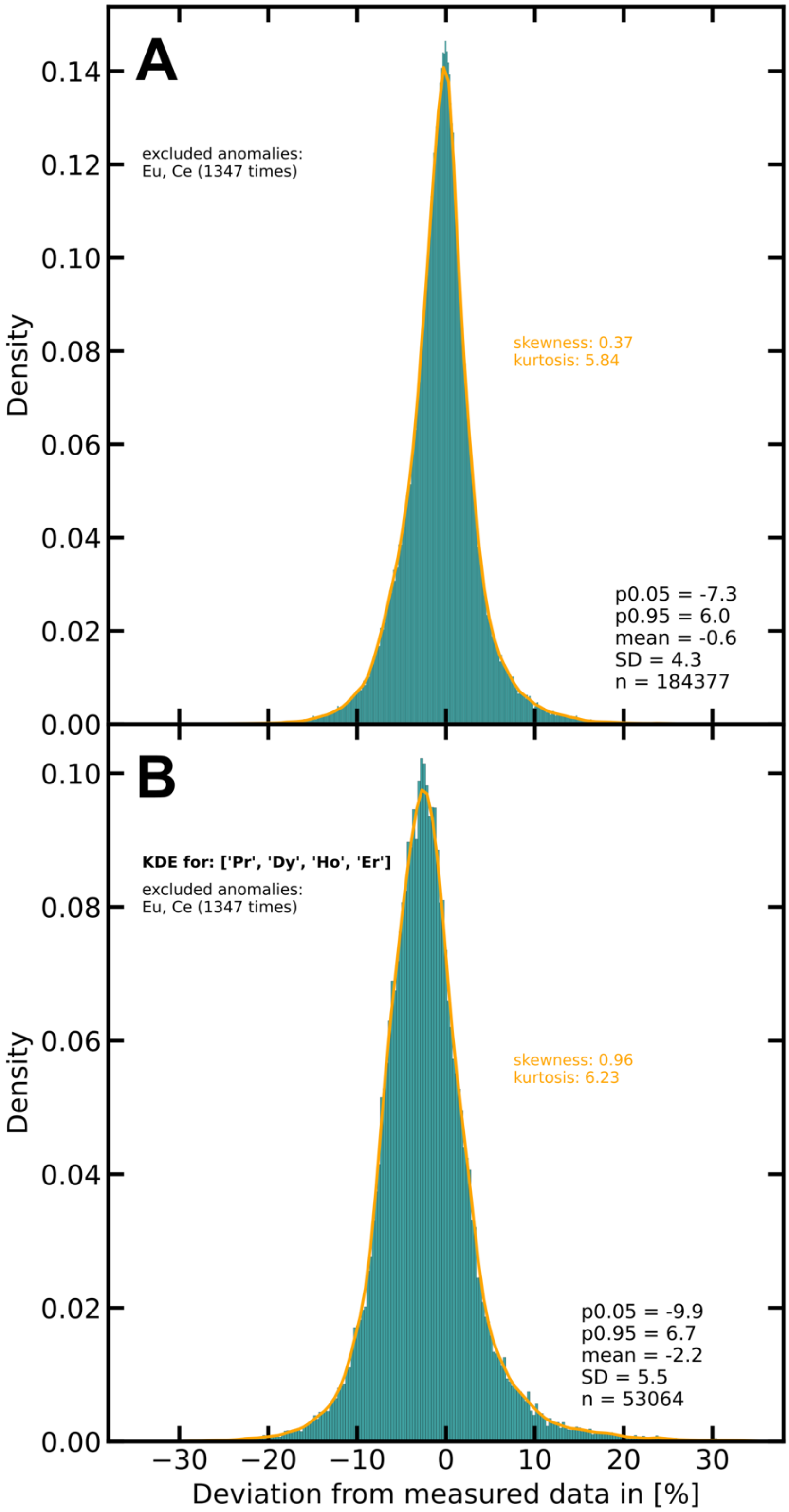

Figure S2: Density histograms that show the deviations of λPM REE from measured REE (NAA-modified) in mafic and ultramafic rock samples from the screened PetDB dataset. The histogram data is normalised to densities. The orange lines give the kernel density estimation curves. Cerium and Eu were excluded 1,292 times as anomalies during modelling. (A) The histogram for all REEs shows a skewness of 0.37, a kurtosis of 5.84. 90 % and the data points range between -7.3 % and 6.0 % (5 % to 95 % percentile; SD=4.3). (B) The histogram for the removed Pr, Tb, Ho and Tm shows a skewness of 0.96, a kurtosis of 6.23. 90 % and the data points range between -9.9 % and 6.7 % (5 % to 95 % percentile; SD=5.5).

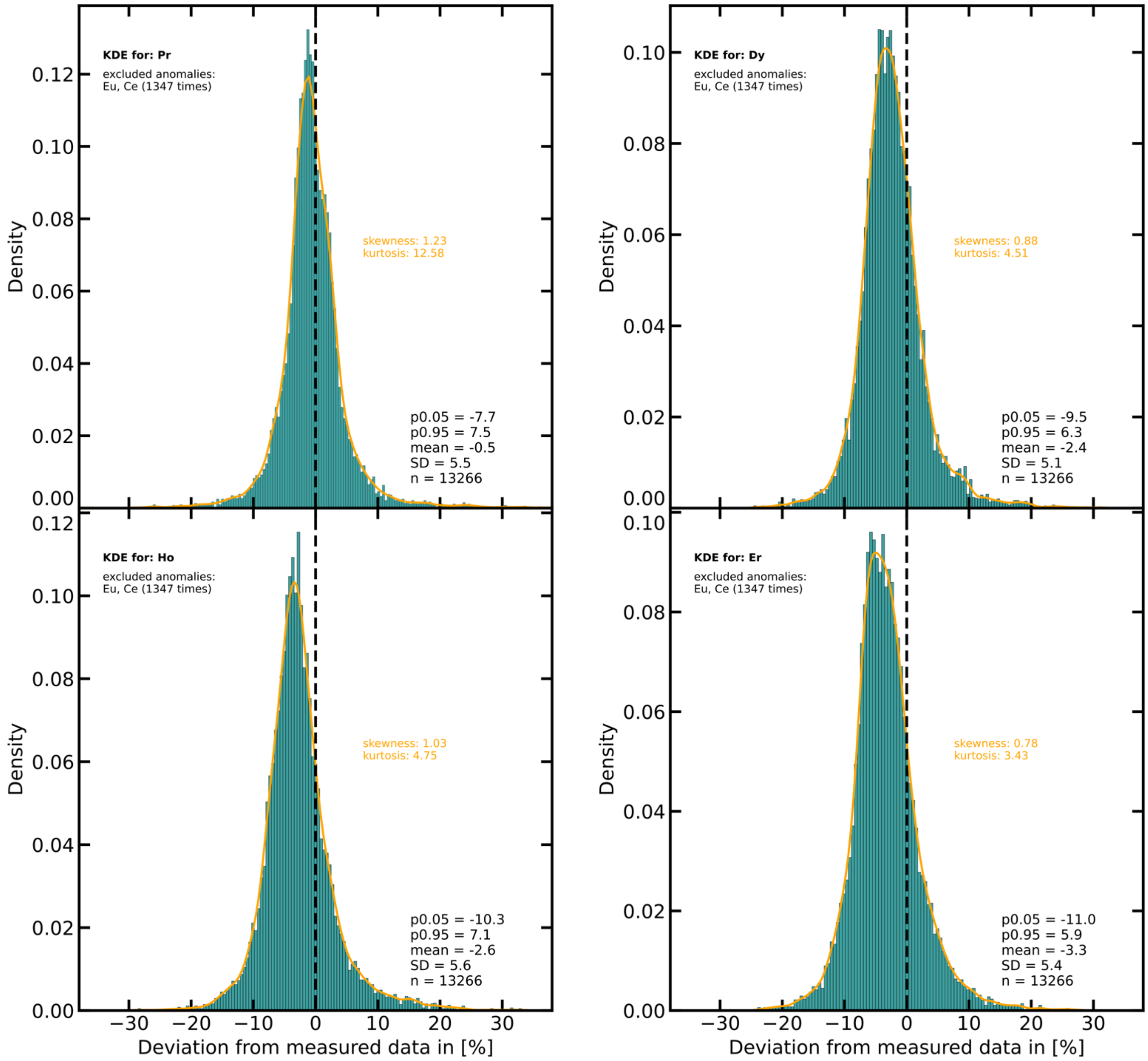

Figure S3: Density histograms that show the deviations of λPM Pr, Dy, Ho and Er from measured data (NAA-modified) in mafic and ultramafic rock samples from the screened PetDB dataset. The histogram data is normalised to densities. While the KDE for Pr is centred at almost zero (mean = -0.5 %), the KDEs for Dy, Ho and Er are slightly shifted to the left with means between -2.4 % and -3.3 %.

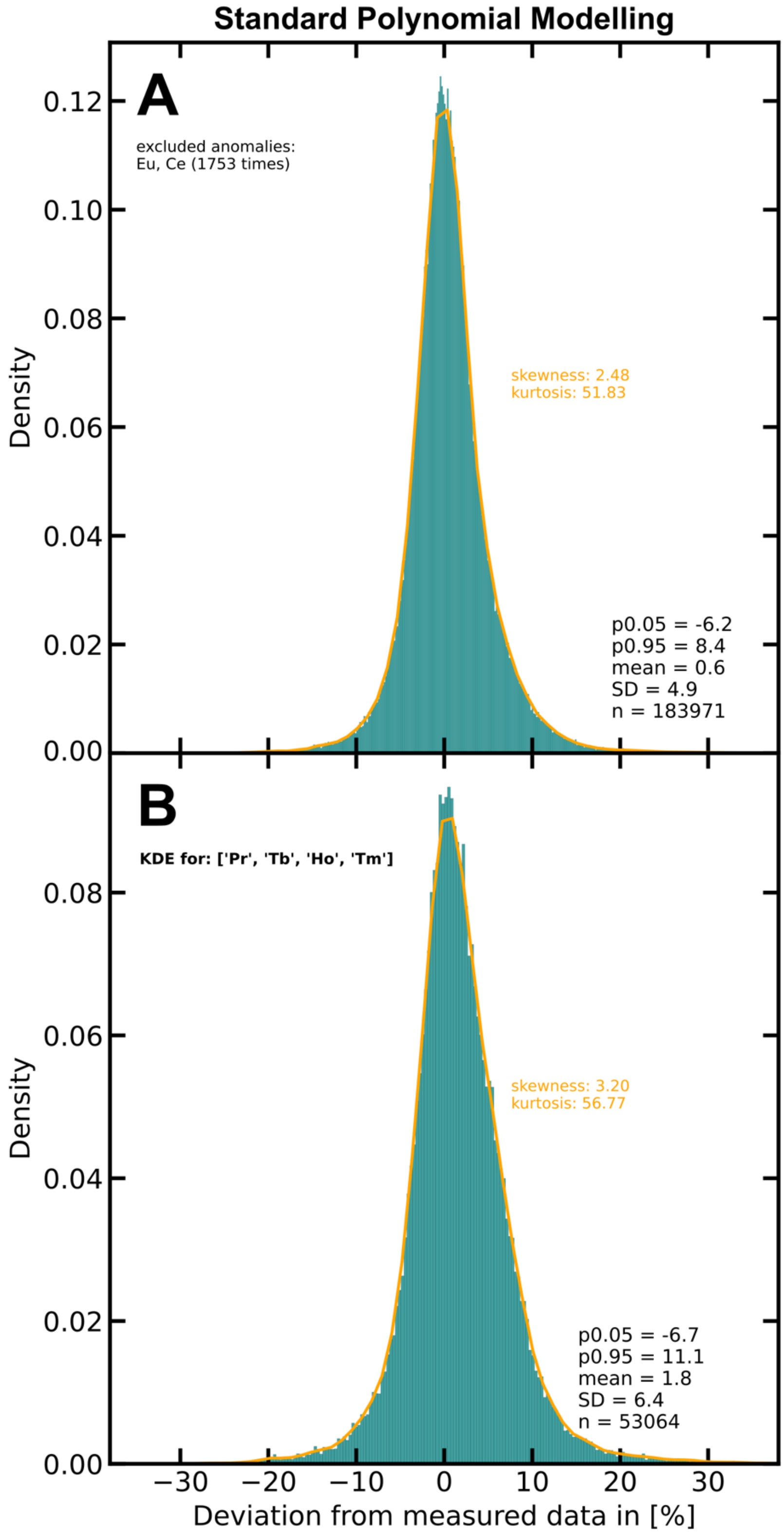

Figure S4: Density histograms that show the deviations of modelled REE (SPM) from measured REE (ID-modified) in mafic and ultramafic rock samples from the screened PetDB dataset (https://search.earthchem.org/). The histogram data is normalised to densities. The orange line gives the kernel density estimation curve. Cerium and Eu were excluded 1,347 times as anomalies during modelling. A) Histogram for all REE that show deviations predominantly between -6.2 % and 8.4 % (5 % to 95 % percentile; SD=4.9). B) Only data for the removed Pr, Tb, Ho and Tm are shown. The deviations predominantly range between -6.7 % and 11.1 % (5 % to 95 % percentile; SD=6.4).

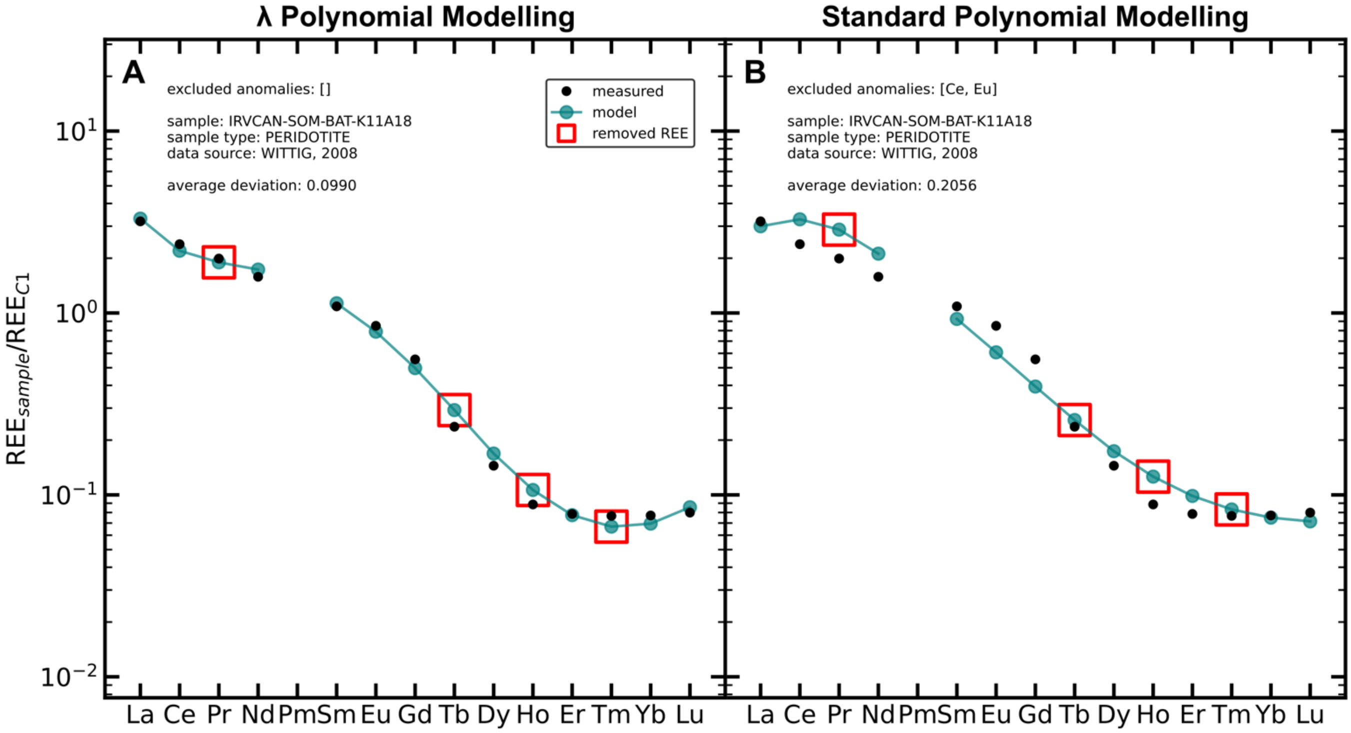

Figure S5: Measured (black) and modelled (green) REE data of a peridotite sample from the PetDB method verification dataset normalised to C1 chondrite [36]. The λPM-modelled data (A) fits the originally measured data more accurately than the PFM-modelled data (B).
